# Delayed egg‐laying and shortened incubation duration of Arctic‐breeding shorebirds coincide with climate cooling

**DOI:** 10.1002/ece3.3733

**Published:** 2017-12-25

**Authors:** Eunbi Kwon, Willow B. English, Emily L. Weiser, Samantha E. Franks, David J. Hodkinson, David B. Lank, Brett K. Sandercock

**Affiliations:** ^1^ Division of Biology Kansas State University Manhattan KS USA; ^2^ Department of Biological Sciences Simon Fraser University Burnaby BC Canada; ^3^ British Trust for Ornithology, The Nunnery Thetford UK; ^4^ Landmark Ecology Ltd Nottingham UK; ^5^ Present address: Department of Fish and Wildlife Conservation Virginia Tech Blacksburg VA USA; ^6^ Present address: Department of Biology Carleton University Ottawa ON Canada; ^7^ Present address: U.S. Geological Survey Upper Midwest Environmental Sciences Center La Crosse WI USA; ^8^ Present address: Norwegian Institute for Nature Research Trondheim Norway

**Keywords:** breeding phenology, *Calidris mauri*, *Calidris pusilla*, climate change, daily nest survival, incubation duration, *Phalaropus lobatus*, seasonality

## Abstract

Biological impacts of climate change are exemplified by shifts in phenology. As the timing of breeding advances, the within‐season relationships between timing of breeding and reproductive traits may change and cause long‐term changes in the population mean value of reproductive traits. We investigated long‐term changes in the timing of breeding and within‐season patterns of clutch size, egg volume, incubation duration, and daily nest survival of three shorebird species between two decades. Based on previously known within‐season patterns and assuming a warming trend, we hypothesized that the timing of clutch initiation would advance between decades and would be coupled with increases in mean clutch size, egg volume, and daily nest survival rate. We monitored 1,378 nests of western sandpipers, semipalmated sandpipers, and red‐necked phalaropes at a subarctic site during 1993–1996 and 2010–2014. Sandpipers have biparental incubation, whereas phalaropes have uniparental incubation. We found an unexpected long‐term cooling trend during the early part of the breeding season. Three species delayed clutch initiation by 5 days in the 2010s relative to the 1990s. Clutch size and daily nest survival showed strong within‐season declines in sandpipers, but not in phalaropes. Egg volume showed strong within‐season declines in one species of sandpiper, but increased in phalaropes. Despite the within‐season patterns in traits and shifts in phenology, clutch size, egg volume, and daily nest survival were similar between decades. In contrast, incubation duration did not show within‐season variation, but decreased by 2 days in sandpipers and increased by 2 days in phalaropes. Shorebirds demonstrated variable breeding phenology and incubation duration in relation to climate cooling, but little change in nonphenological components of traits. Our results indicate that the breeding phenology of shorebirds is closely associated with the temperature conditions on breeding ground, the effects of which can vary among reproductive traits and among sympatric species.

## 1 INTRODUCTION

Effects of contemporary climate change have been found across all biomes as organisms shift their seasonal phenologies and geographic distributions (Parmesan & Yohe, 2003). The ecological fingerprints of climate change are also found in species interactions, community shifts, pest and disease dynamics, and species extinctions (Cahill et al., 2013; McCarty, 2001; Parmesan, 2006). Advancement in the timing of breeding among bird populations has been one of the clearest indicators of organismal responses to climate change (Both et al., 2004; Brown, Li, & Bhagabati, 1999; Crick, Dudley, Glue, & Thomson, 1997; Dunn & Winkler, 1999; McCarty, 2001; McCleery & Perrins, 1998; Slater, 1999). Timing of breeding is often selected to maximize either the survival of breeders or their productivity (Perrins, 1970; Te Marvelde, Webber, Meijer, & Visser, 2011). Shifts in breeding phenology may be due to microevolutionary change or phenotypic plasticity that provides breeding birds with an adaptive buffer under a variable or changing climate (Charmantier et al., 2008).

Birds can not only vary their timing of breeding, but also vary egg volume or yolk content, egg‐laying interval, clutch size, nest attentiveness, and/or duration of incubation to maximize reproductive success under variable environmental conditions (Hargitai et al., 2016; Martin, 2002; Royle, Hartley, & Parker, 2006). Typically, breeding earlier in the season is associated with higher reproductive output, and seasonal declines are commonly found in clutch size (Crick, Gibbons, & Magrath, 1993; Stenseth & Mysterud, 2002), egg size (Birkhead & Nettleship, 1982; Hipfner, Gaston, & Gilchrist, 2005), and the number or quality of fledglings (Lepage, Gauthier, & Menu, 2000; Grant, Shaffer, Madden, & Pietz, 2005; García Borboroglu, Yorio, Moreno, & Potti, 2008). These declines can arise due to direct or indirect effects of seasonal timing and/or differences in the quality of breeders (Verhulst & Nilsson, 2008). A direct link between the timing of breeding and reproductive performance can be viewed as a proximate response to current conditions regardless of calendar dates, or a response to date per se, with date‐specific strategies driven by, for example, the time remaining to the end of food availability for chicks (Lank, Oring, & Maxson, 1985), or probability of successful fall migration for adults (Jamieson, Ydenberg, & Lank, 2014; Lank, Butler, Ireland, & Ydenberg, 2003).

**Figure 1:**
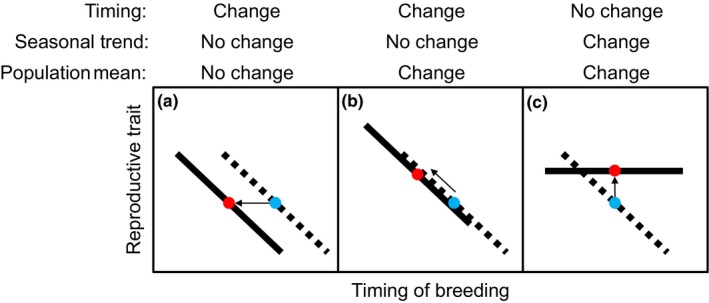
Hypothesized relationships between the timing of breeding and a reproductive trait under three different scenarios with changing temperature and its effect on the timing of breeding. The simplified graphs show a hypothetical trait which is negatively correlated with the timing of breeding, and possible advancement in the timing of breeding between two time periods with different conditions: warmer (mean = red dot, trend = solid line) and cooler (mean = blue dot, trend = dotted line). (a) Population mean of a trait did not change after the timing of breeding advanced. (b) Population mean increased as the timing of breeding advanced and the seasonal trend remained consistent. (c) Timing of breeding did not change, but the population mean increased as the seasonal trend disappeared. The last scenario may occur if favorable conditions under a warming climate eliminate seasonal constraints and hence the seasonal trend. Direction of arrows will be reversed in the case of climate cooling. After Winkler et al. (2002)

As the timing of breeding advances due to a warming climate, one might expect long‐term changes in the within‐season relationships between timing of breeding and reproductive traits, changes in the mean value of reproductive traits, or both (Figure 1). Given that clutch size is typically larger for earlier breeders, Winkler, Dunn, and McCulloch (2002) hypothesized that climate‐induced long‐term advancement in egg‐laying would increase mean clutch size in birds. In tests with tree swallows (*Tachycineta bicolor*), the authors did not detect the expected increase in clutch size and concluded that the maximum clutch size of early breeders is constrained by factors other than the laying date. Few studies have tested for additional impacts of long‐term changes in climatic conditions on reproductive traits beyond the timing of egg‐laying (Both & Visser, 2005; Dickey, Gauthier, & Cadieux, 2008; Skagen & Adams, 2012), and most have considered northern temperate species of birds with flexible reproductive traits, such as a variable clutch size or multiple breeding attempts per season (but see Skinner, Jefferies, Carleton, & Abraham, 1998). There is a need to test for climate‐induced long‐term changes in a suite of reproductive traits in other regions of the globe.

Over recent decades, regions at high latitudes have exhibited a faster rate of climate change than anywhere else on the globe, including warming temperatures and earlier snow melt, with rapid changes expected to continue into the future (AMAP, 2012; IPCC, 2014). In some areas of the Arctic, birds from small‐bodied sandpipers to large‐bodied geese have advanced the timing of first egg‐laying (Clausen & Clausen, 2013; Liebezeit, Gurney, Budde, Zack, & Ward, 2014; McKinnon, Picotin, Bolduc, Juillet, & Bêty, 2012). However, several studies have also shown no significant changes in breeding phenology of birds in the Arctic (Høye, Post, Meltofte, Schmidt, & Forchhammer, 2007; Mortensen, Schmidt, Høye, Damgaard, & Forchhammer, 2016; Reneerkens et al., 2016). Only a few studies have investigated how other reproductive traits are subsequently impacted by changes in the timing of breeding in response to climate change in the Arctic (Gurney et al., 2011). Given that fecundity is affected by a suite of reproductive traits such as clutch size, egg volume, and daily nest survival, it is important to understand how climate‐induced long‐term changes in the breeding phenology affect these traits.

The objective of our project was to quantify long‐term changes in breeding performance of three species of sympatric shorebirds in an Arctic region that is experiencing rapid climate change. We investigated long‐term changes in the timing of breeding and seasonal variation in the reproductive traits of three migratory shorebird species, western sandpipers *Calidris mauri*, semipalmated sandpipers *C. pusilla*, and red‐necked phalaropes *Phalaropus lobatus*, between two study periods that spanned two decades. Timing of clutch initiation is often predicted with the temperature conditions experienced immediately before and during the egg‐laying stage (Crick & Sparks, 1999; Ockendon, Leech, & Pearce‐Higgins, 2013). We first tested whether (1) the timing of clutch initiation correlates with the spring temperature during prelaying or laying stages on site. Based on the general warming trend at high latitudes, we then predicted that (2) spring temperatures during prelaying or laying stages would be warmer and the timing of shorebird clutch initiation would advance from the 1990s to the 2010s. Within‐season declines have been documented for clutch size, egg volume, and hatching success of two species in our study populations (Sandercock, 1998; Sandercock, Lank, & Cooke, 1999). Given the known links between the timing of breeding and reproductive performance, we predicted that (3) advances in clutch initiation in response to warmer temperatures would be coupled with increases in mean clutch size, egg volume, and nesting success (Figure 1b). Warming temperatures may change the magnitude of within‐season trends of reproductive traits (Figure 1c). Therefore, we also tested whether (4) the relationships between clutch initiation date and subsequent reproductive traits have changed between decades.

## 2 MATERIALS AND METHODS

### 2.1 Study site

**Figure 2:**
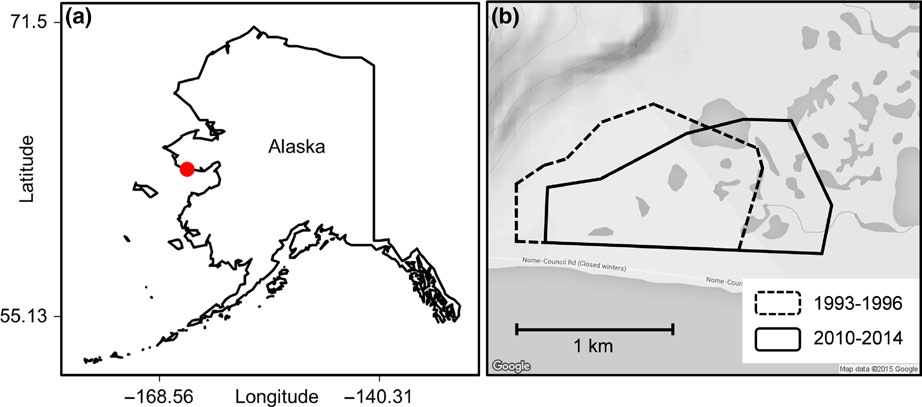
Location of study site at Nome, Alaska (a), and boundaries of the study plots during two study decades (b). Darker shades in (b) indicate both fresh and marine water bodies

A 4‐km^2^ study plot was set up in 1993 near Cape Nome, 21 km east of the town of Nome (64°20′N, 164°56′W) on the Seward Peninsula of western Alaska (Figure 2; Sandercock, 1998). The plot was composed of lowland tundra with small ridges and tussocks, interspersed with ponds containing fresh and brackish water from a channel connected to the Bering Sea. Located just below the Arctic Circle and on the coast of the Bering Sea, the field site experiences an Arctic climate. We monitored shorebird breeding ecology during two study decades separated by 15 years (1993–1996, hereafter “1990s,” and 2010–2014, hereafter “2010s”) using standardized field protocols (English, 2014; Sandercock, 1997a,b, 1998).

### 2.2 Study species

Three species of migratory shorebirds, western sandpipers, semipalmated sandpipers, and red‐necked phalaropes, breed sympatrically at our study plot. After arrival at the breeding area in Nome, shorebirds are faced with a short Arctic summer, with a breeding and brood‐rearing window that spans ~2 months, from mid‐May to mid‐July. The three species show a modal clutch size of four eggs and have precocial young capable of self‐feeding immediately after hatching (Sandercock, 1997b, 1998). The two species of sandpipers are socially monogamous with biparental incubation and brood‐rearing largely by males (Franks, David, & Herbert, 2014; Hicklin & Gratto‐Trevor, 2010). In contrast, red‐necked phalaropes have a polyandrous mating system with uniparental male incubation and brooding (Sandercock, 1997b; Schamel, 2000; Schamel, Tracy, & Lank, 2004). Reproductive traits of the two sandpiper species, such as clutch size, egg volume, and hatching success, exhibited within‐season declines over the course of the season in our study populations (Sandercock, 1998; Sandercock et al., 1999). Although not previously studied at our field site, red‐necked phalaropes exhibited a within‐season increase of egg size and decrease in incubation duration at other subarctic sites in western Alaska and Canada (Reynolds, 1987; Schamel, 2000; Schamel et al., 2004).

### 2.3 Field data collection

We estimated clutch initiation dates using backdating from stage of incubation as determined by egg flotation (Liebezeit et al., 2007; Sandercock, 1998). For every nest we found, egg length (*L*) and breadth (*B*) were measured to 0.1 mm, and egg volume was calculated using the formula *V* = *kLB* ^2^, where *k* = 0.47 for the pyriform eggs of shorebirds (Governali, Gates, Lanctot, & Holmes, 2012; Sandercock, 1998). For the subset of nests found during egg‐laying and monitored until hatching, we calculated the length of the incubation period from the day that the last egg was laid to the day the first egg hatched (*N* = 34 nests during 1990s and 89 nests during 2010s; sample sizes for each species are shown in Table 1).

**Table 1:** Breeding parameters of three species of migratory shorebirds during the 1990s (1993–1996) and 2010s (2010–2014) at Nome, Alaska

Response	1990s			2010s			Decadal diff.
	Mean (*N*)	LCI	UCI	Mean (*N*)	LCI	UCI	
***(a) Date of clutch initiation***							
Western sandpiper	May 24 (225)	May 21	May 26	May 28 (309)	May 22	Jun 2	+4.3
Semipalmated sandpiper	May 28 (202)	May 25	May 30	Jun 2 (250)	May 26	Jun 6	+4.8
Red‐necked phalarope	Jun 3 (56)	May 31	Jun 5	Jun 8 (293)	Jun 1	Jun 12	+4.8
***(b) Clutch size (no. eggs)***							
Western sandpiper	3.86 (227)	3.78	3.94	3.73 (326)	3.54	3.91	−0.13
Semipalmated sandpiper	3.76 (207)	3.68	3.85	3.78 (259)	3.59	3.98	+0.02
Red‐necked phalarope	3.87 (62)	3.73	4.01	3.71 (297)	3.41	4.00	−0.16
**(c) Egg volume (** ***ml*** **)**							
Western sandpiper	7.16 (161)	7.03	7.28	7.09 (269)	6.80	7.19	−0.07
Semipalmated sandpiper	6.30 (181)	6.18	6.42	6.35 (220)	6.09	6.63	+0.05
Red‐necked phalarope	5.89 (61)	5.67	6.12	5.88 (214)	5.36	6.40	−0.01
***(d) Incubation duration (days)***							
Western sandpiper	22.9 (19)	22.4	23.3	20.7 (47)	19.7	21.7	−2.2
Semipalmated sandpiper	22.0 (10)	21.2	22.7	20.1 (25)	18.5	21.6	−1.9
Red‐necked phalarope	19.6 (5)	18.4	20.7	21.5 (17)	19.1	23.9	+1.9
***(e) Daily nest survival***							
Western sandpiper	0.971 (193)	0.963	0.977	0.964 (248)	0.939	0.979	−0.007
Semipalmated sandpiper	0.958 (176)	0.949	0.967	0.965 (169)	0.943	0.980	0.007
Red‐necked phalarope	0.953 (45)	0.929	0.968	0.937 (151)	0.856	0.973	−0.016

Estimates for differences between decades are from mixed‐effects models with a fixed effect of study decade and a random effect of year on the intercept. *N*, sample size of nests. 95% confidence interval is shown for each estimate.

Daily mean temperature data were available from a meteorological station at the Nome Airport, ~25 km west of the study plot (64°31′N, 165°26′W). Daily mean temperature was also measured at the study plot during the breeding season of 2010–2014 using an automated portable weather station (Onset Hobo Weather Station, U30 Series; Pocasset, Massachusetts, USA) and was strongly correlated with the temperature measured at the meteorological station (*r* ^2^ = .84, *p *> .001). Therefore, we used the daily weather data collected at Nome Airport to represent the climatic conditions at our study plot during both decades.

### 2.4 Data analysis

#### 2.4.1 Effects of weather on timing of breeding

We modeled dates of clutch initiation as a function of fixed effects of species and five abiotic covariates (average daily mean temperature and average daily total precipitation of species‐specific prelaying window and the egg‐laying window, and maximum snow accumulation during the prior winter), with a random effect of year. We defined the egg‐laying window for each species as the mean date of clutch initiation ±2 *SD* for all years combined, and the species‐specific prelaying window as the 2 weeks prior to each species’ egg‐laying window. Daily mean temperature and daily total precipitation were averaged over the species‐specific prelaying and laying windows for each year. We standardized covariates by subtracting the mean and dividing by 2SD and checked for multicollinearity using variance inflation factor <5 as our cutoff value (Gelman & Su, 2015; O'Brien, 2007). We used Akaike Information Criteria corrected for small sample sizes (AIC_*c*_) to compare 64 submodels (Burnham & Anderson, 2002; Table S1). Models with ΔAIC_*c*_ ≤ 2 were considered as candidates for the top model set and were model‐averaged using the natural average method (Burnham & Anderson, 2002; Nakagawa & Freckleton, 2011).

#### 2.4.2 Long‐term changes of climatic drivers

We quantified a 22‐year change (1993–2014) in daily mean temperature by fitting a linear model to each day of a year, with each day's mean climatic value as the response and year as the predictor. We used the slope of this model as an index of long‐term change for each day of the year. To test whether average daily mean temperatures during the prelaying and laying windows have significantly changed between 1993–1996 and 2010–2014, we fitted linear mixed‐effects models (LMMs) to the daily mean temperature of the prelaying and laying windows separately, with decade and date as fixed effects, and year as a random effect, using the “lme” function of the R package *nlme* (Pinheiro, Bates, DebRoy, & Sarkar, 2017; R Development Core Team, 2016).

#### 2.4.3 Within‐season patterns in breeding performance

We used analysis of covariance (ANCOVA) to test for within‐season variation in clutch size, mean egg volume per clutch, incubation duration, and daily nest survival rate. Prior to performing analyses, we subtracted the annual mean date of clutch initiation from the clutch initiation date of each nest to center the dates to the population mean for each species. Centering allowed us to separate the effects of breeding earlier than other birds within each season from the effects of breeding on a certain day of the year. As most birds in our population lay four‐egg clutches (Sandercock, 1998; Sandercock et al., 1999), we treated clutch size as a binomial variable with two levels, ≤3 eggs versus 4 eggs in a clutch. To model clutch size, we fit a generalized linear mixed model (GLMM) with a binomial distribution (logit link) for each species, using the “glmer” function of the R package *lme4* (Bates, Maechler, Bolker, & Walker, 2014). Distributions of mean egg volume and incubation duration were normal, so we used LMMs for analyses of those reproductive traits. Each model included three fixed effects: centered date of clutch initiation as a continuous variable, study decade as a categorical variable, and the interaction between date of clutch initiation and decade. We included a random effect of year on both the intercept and the slope for the effect of the date of clutch initiation. We concluded that the breeding parameter showed a within‐season pattern if the effect of clutch initiation date was significant based on whether the 95% confidence interval of the effect size overlapped zero. For each species, we estimated daily nest survival rates (DSR) using the nest survival model in R package *RMark* (Laake, 2013; White & Burnham, 1999). Prior to analysis, we excluded known renests as well as a subset of 249 nests that were subjected to different experimental manipulations which could have affected nest survival (English et al. 2014; Sandercock, 1997a,b). For DSR, the same three fixed effects were tested using *RMark* which did not allow testing for a random effect of year.

#### 2.4.4 Decadal changes in within‐season patterns of breeding performance

From the ANCOVA with three fixed effects (centered date of clutch initiation, study decade, and the interaction between date of clutch initiation and decade), we concluded that the within‐season pattern of a given reproductive trait changed between the 1990s and 2010s if the interaction term was significant. We considered the interaction to be significant if the 95% confidence interval of the effect sizes did not overlap zero. For DSR, we tested the same three fixed effects without any random effect using *RMark*.

#### 2.4.5 Decadal changes in average breeding parameters

To test for apparent decadal changes in the breeding performance of shorebirds, we tested for an effect of study decade (1990s vs. 2010s) on timing of clutch initiation, clutch size, mean egg volume per clutch, and incubation duration, including a random effect of year. We fit LMMs to the mean egg volume per clutch and observed incubation duration, and a GLMM to clutch size. We compared the DSR of each species between the two decades by constructing a model with decade as the sole explanatory variable. Average nest success over a breeding season was calculated as the product of estimated DSR for an exposure period that included egg‐laying (4 days) plus the incubation duration that we calculated from a subset of nests per decade for each species (ca. 20–23 days, see Table 1). Estimates of variance and standard error for extrapolated estimates of nest survival were calculated using the delta method (Powell, 2007). We then compared the 95% confidence intervals of nest survival rates to test for a significant difference in DSR between decades.

## 3 RESULTS

### 3.1 Effects of weather on timing of breeding

**Figure 3:**
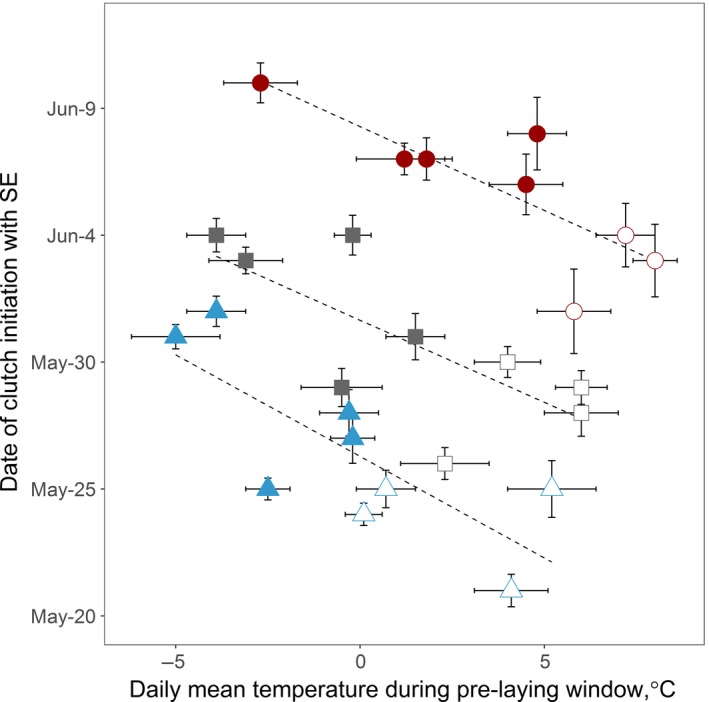
Relationship between daily mean temperature during the species‐specific prelaying window and the mean date of clutch initiation per species per year. Circle: red‐necked phalarope, square: semipalmated sandpiper, triangle: western sandpiper. Filled: 2010–2014, open: 1993–1996. Dashed lines show simple linear relationships

We monitored 496 shorebird nests during the 1990s and 882 nests during the 2010s. Average daily mean temperatures during both the prelaying and the egg‐laying windows were strongly negatively correlated with the timing of clutch initiation (β_prelay_ = −4.2, 95% CI = −4.9 to −3.5; β_lay_ = −3.1, 95% CI = −3.8 to −2.4; Table 2). Based on the averaged model, a 1°C decrease in the average daily mean temperature during the species‐specific prelaying window and laying window delayed clutch initiation by 0.6 and 0.8 days, respectively (Table 2). The relationships between the average daily mean temperature during prelaying windows and the mean date of clutch initiation were similar among the three species (Figure 3).

**Table 2:** Model‐averaged effect sizes of species and climate covariates on the timing of clutch initiation at Nome, Alaska, 1993–1996 and 2010–2014

	Estimate	*SE*	LCI	UCI
(Intercept)	145.2	0.3	144.7	145.7
Semipalmated sandpiper	5.5	0.4	4.8	6.3
Red‐necked phalarope	14.4	0.5	13.5	15.3
Prelaying temperature	−4.2	0.4	−4.9	−3.5
Laying temperature	−3.1	0.4	−3.8	−2.4
Rain during prelaying	−0.1	0.4	−0.9	0.6
Rain during laying	−0.2	0.4	−1.0	0.6

Covariates included in the global model were average daily mean temperature and average daily total precipitation during the prelaying and the egg‐laying stages, the maximum snow accumulation during the prior winter, and species (base group: western sandpiper), with year as a random effect. Covariates were standardized by centering on the mean and dividing by 2 *SD*. The full model set is given in Table S1. Standard error and 95% confidence interval are shown for each estimate. Western sandpiper, *N* = 533 nests; semipalmated sandpiper, *N* = 451 nests; red‐necked phalarope, *N* = 347 nests.

### 3.2 Long‐term changes in climatic drivers and timing of breeding

**Figure 4:**
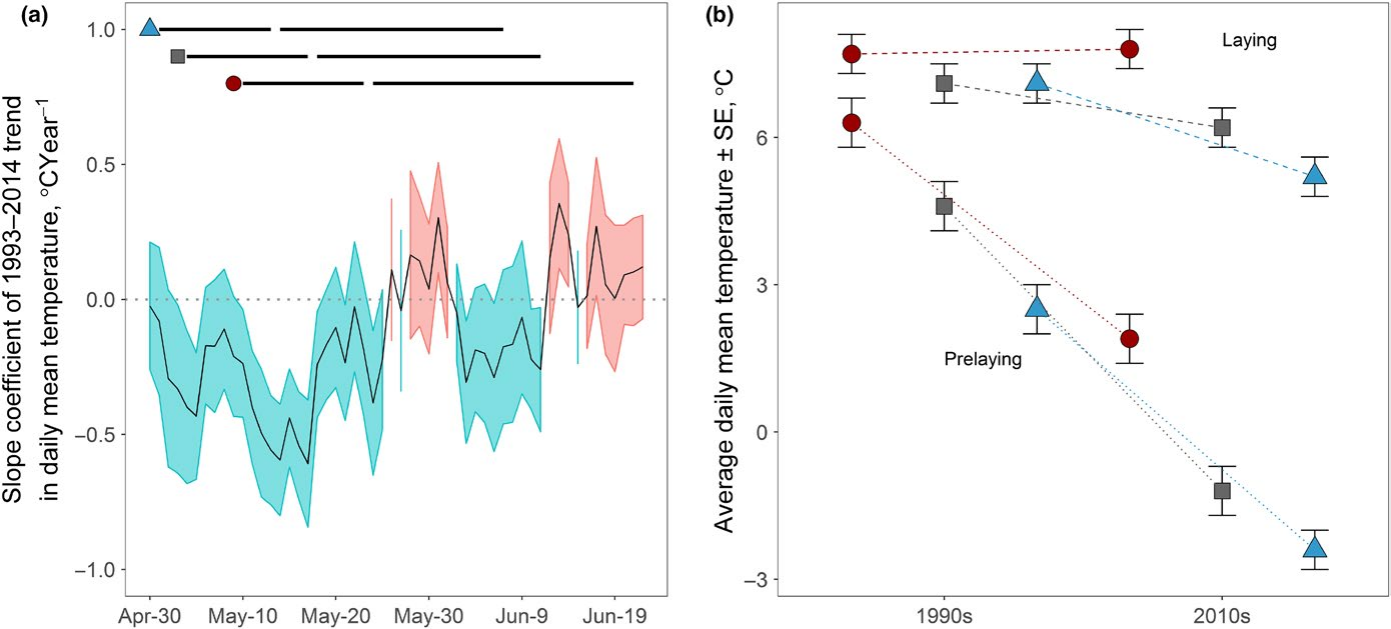
Long‐term trends in daily mean temperature over the 22‐year period of 1993–2014 (a) and decadal changes in mean temperature during the species‐specific windows of prelaying and laying (b) at Nome, Alaska. (a): Slope coefficients (black line) are shown with ±standard error. Cooling trends are shown in blue (slope coefficient < 0), and warming trends are shown in red (slope coefficient > 0). Horizontal lines in the panel (a) show the span of egg‐laying window for each species defined as the mean date of clutch initiation ±2 *SD* and the prelaying window defined as 2 weeks prior to egg‐laying window for all years combined. Circle: red‐necked phalarope, square: semipalmated sandpiper, triangle: western sandpiper. (b): Daily mean temperatures during prelaying and egg‐laying windows are averaged across 1993–1996 for 1990s and 2010–2014 for 2010s

**Figure 5:**
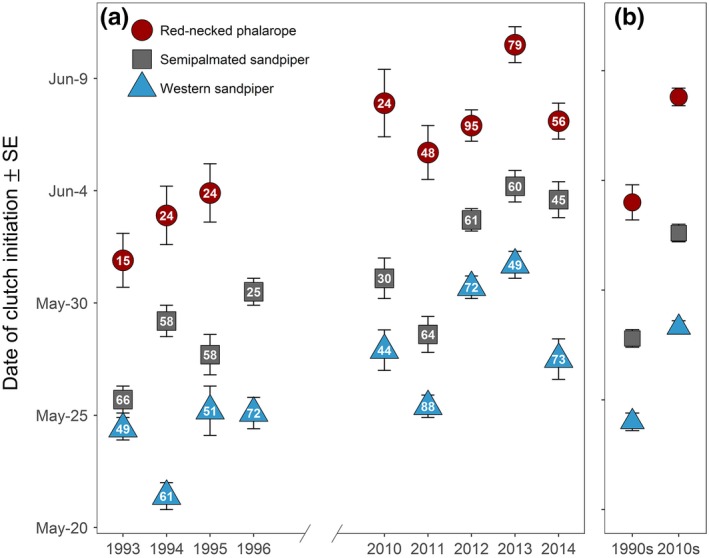
Dates of clutch initiation for three shorebird species at Nome, Alaska, 1993–1996 and 2010–2014, shown by year (a) and by decades (b). Values inside points are the number of monitored nests

A mix of warming and cooling trends across a 22‐year period (1993–2014) was observed for different days of the year (Figure 4a). A cooling trend occurred during the prelaying windows of all species, resulting in daily mean temperatures that averaged 4.9, 5.8, and 4.4°C cooler during the 2010s than the 1990s for western sandpiper, semipalmated sandpiper, and red‐necked phalaropes, respectively (Figure 4a,b). Both warming and cooling trends occurred within the laying windows of the three shorebird species, and as a result, the daily mean temperatures during the laying windows did not differ significantly between the 1990s and 2010s (−1.1 ± 1.3°C, *t* = −0.8, *p* = .43; Figure 4a,b). Clutch initiation was 4.3–4.8 days later in the 2010s than 1990s in all three species (Table 1; Figure 5a,b).

### 3.3 Within‐season patterns in breeding performance

**Figure 6:**
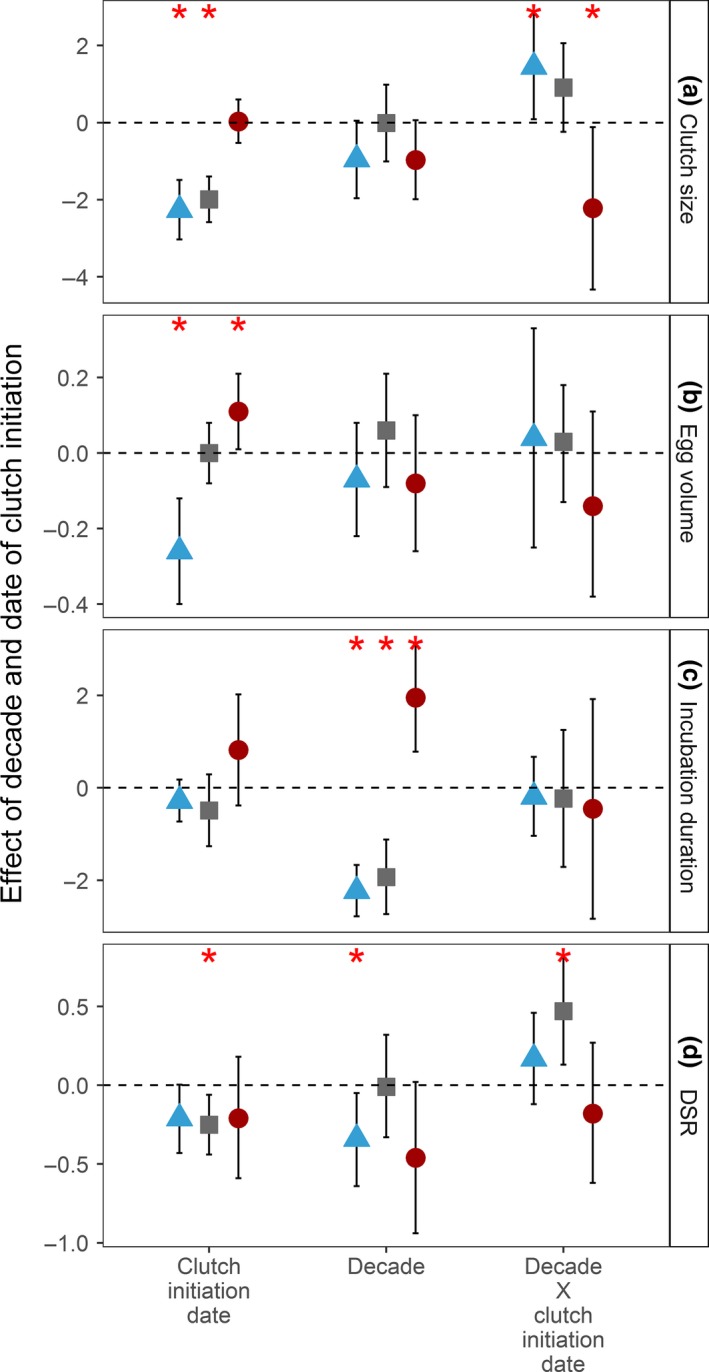
Effect sizes of date of clutch initiation, study decade (1990s vs. 2010s), and their interaction with 95% confidence intervals on the breeding parameters of shorebirds at Nome, Alaska, 1993–1996 and 2010–2014. Models included a random effect of year on the intercept and on the slope. A random effect on the slope is equivalent to an effect of laying date. A generalized linear mixed model was fit to the probability of laying a four‐egg clutch, and linear mixed models were fit for egg volume and incubation duration. For daily nest survival rates, fixed‐effect models were fit without random effects. Asterisks indicate significant effects where the 95% CIs do not overlap zero. Samples sizes are given in Table 1

**Figure 7:**
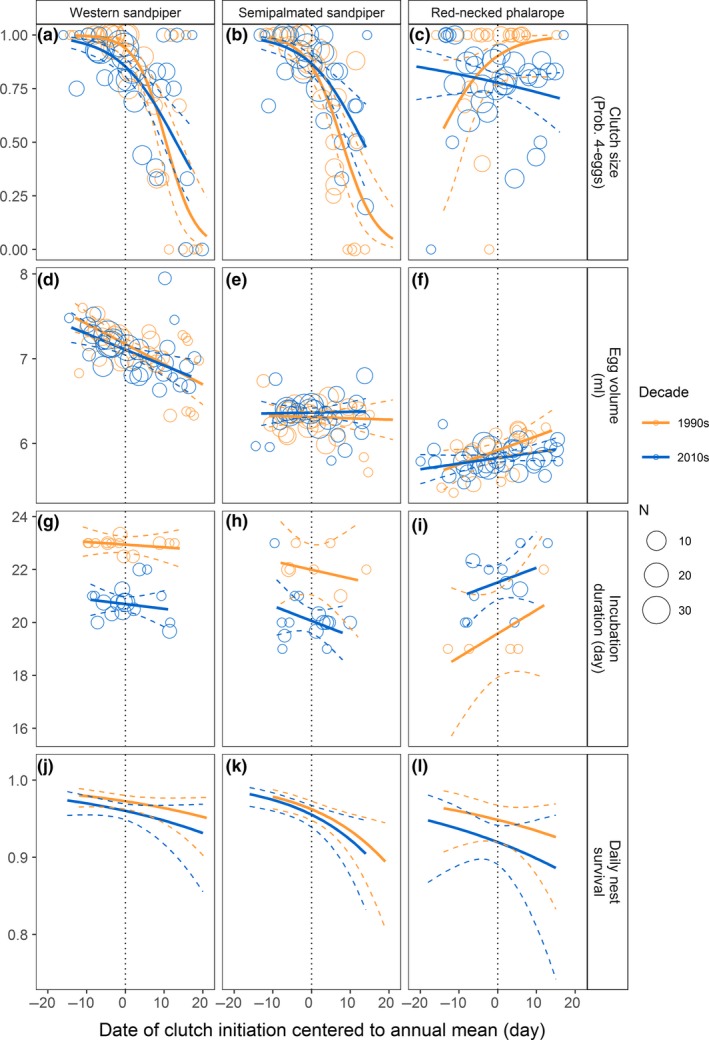
Within‐season patterns in the clutch size, egg volume, incubation duration, and daily nest survival of shorebirds at Nome, Alaska, monitored during 1993–1996 and 2010–2014. Within‐season patterns are shown for each decade (solid line, orange: 1990s; blue: 2010s) with 95% confidence intervals (dashed line). Points represent the daily mean values for breeding parameters during the 1990s (orange) and 2010s (blue). Size of each point reflects the sample size (*N*). Daily nest survival rates were estimated values. The laying date of each nest was centered on the mean clutch initiation date for the corresponding year (represented by the dotted vertical line). Thus, negative values indicate that clutch initiation was earlier than the population mean of a species in a year

The clutch size of four eggs occurred in 81% of 1,378 nests of three species combined. The probability of having a clutch with fewer than four eggs was greater for nests initiated later in the season for the two sandpiper species, but not for phalaropes (Figures 6a and 7a–c). Egg volume showed a strong within‐season decline in western sandpipers, no trend in semipalmated sandpipers, and an increasing trend in red‐necked phalaropes (Figures 6b and 7d–f). There was no within‐season variation in incubation duration for any species (Figures 6c and 7g–i). We determined nest fate for 414 nests during the 1990s and 568 nests during the 2010s. Within‐season declines in the daily nest survival rates occurred in all three species and were statistically significant for semipalmated sandpipers (Figures 6d and 7j–l).

### 3.4 Decadal changes in within‐season patterns of breeding performance

We did not detect significant changes in the within‐season patterns in breeding performance between the 1990s and 2010s for most reproductive parameters (Figure 6). However, the within‐season decline of clutch size tended to be steeper in the 2010s than in the 1990s for western sandpipers (Figures 6a and 7a). On the other hand, the relationship between the date of clutch initiation and the clutch size of red‐necked phalaropes was positive during the 1990s, but then negative during the 2010s (Figures 6a and 7c). In semipalmated sandpipers, the slope of the seasonal decline in DSR was greater for 1990s than 2010s (Figures 6d and 7k).

### 3.5 Decadal changes in average breeding parameters

The mean clutch size and egg volume did not change between the 1990s and 2010s for any species. Mean daily nest survival rates (DSR) decreased only in western sandpipers (95% CI, 1990s: 0.963–0.977, 2010s: 0.939–0.979; Table 1, Figure 6d). Incubation duration was calculated from fewer nests than other reproductive traits, especially for the 1990s, during which we documented the beginning of incubation through hatching for only 19, 10, and 5 nests of western sandpiper, semipalmated sandpiper, and red‐necked phalaropes, respectively (Table 1). Despite small sample sizes, the mean incubation duration significantly decreased for western sandpipers and semipalmated sandpipers by −2.2 and −1.9 days, respectively, but increased by +1.9 days for red‐necked phalaropes between the 1990s and 2010s (Figures 6c and 7g–i). The expected probability of a nest surviving to hatch, based on DSR and the observed length of the incubation period, was not different from the 1990s for semipalmated sandpipers (95% CI, 1990s: 0.39–0.55, 2010s: 0.32–0.46) or for western sandpipers (95% CI, 1990s: 0.29–0.44, 2010s: 0.31–0.48), but tended to be lower in the 2010s for red‐necked phalaropes (95% CI, 1990s: 0.16–0.49, 2010s: 0.08–0.19).

## 4 DISCUSSION

Our study of climate change and breeding performance of Arctic‐breeding shorebirds resulted in three major findings. First, despite overall warming trends for the Arctic, a long‐term cooling trend occurred during the early part of the breeding season at Nome, Alaska. Second, in an apparent response to this cooling trend, all three species of shorebirds delayed egg‐laying by ~5 days during the 2010s compared to the 1990s. Last, changes in breeding phenology between decades did not lead to changes in mean clutch size, egg volume, or daily nest survival rate of the population. However, the incubation duration, which showed little intraseasonal variation, changed between the decades for all three species of shorebirds.

### 4.1 Climatic trends and delayed laying

The prelaying window of shorebirds was unexpectedly cooler in the 2010s than the 1990s. It is well established that the rate of climate change can vary among different seasons of the year (AMAP, 2012; Doran et al., 2002). However, we showed that both cooling and warming trends at Nome occurred within the month‐long laying window of shorebirds. Despite the short length of the Arctic summer, a single annual metric pinpointed on a certain date or even averaged across the season might not adequately represent temporal changes in the temperature‐related cues that mediate the phenology of Arctic organisms. Our results suggest the importance of examining climatic conditions at a range of different temporal scales surrounding the timing of ecological processes.

The timing of clutch initiation in shorebirds was delayed in recent decades and was correlated with cooler temperatures during the prelaying and laying windows. Although not as prevalent as climate warming, ecological fingerprints of climate cooling are found across many biomes (Doran et al., 2002; Parmesan & Yohe, 2003). Delayed laying at our site could have resulted from (1) delayed arrival to the breeding grounds, (2) colder temperatures limiting adult food availability and/or snow‐free nesting habitat, which can constrain egg formation and/or laying, (3) a direct consequence of lower temperatures on metabolic rates, or even (4) an adaptive phenological shift to match the timing of hatching with local food peaks, which might also be delayed with cooler temperatures (Tulp & Schekkerman, 2008). The interval between dates when two sandpiper species arrived on the study plot and initiated their clutch was shorter in the 2010s than in the 1990s, which indicates that the colder temperature in the 2010s is unlikely to have constrained egg formation in our population (E. Kwon, unpublished data). Further study is needed to separate the effects of delayed arrival from the effects of local environmental conditions on the timing of breeding.

### 4.2 Within‐season patterns and decadal changes of breeding parameters

We found within‐season declines in clutch size of both sandpiper species, egg volume of western sandpipers, and daily nest survival rate of semipalmated sandpipers, consistent with patterns previously reported for the two sandpiper species (Johnson & Walters, 2008; Nol, Blanken, & Flynn, 1997; Ruthrauff, 2002; Sandercock et al., 1999). Within‐season increases in egg volume were also consistent with previous observations for red‐necked phalaropes (Hildén & Vuolanto, 1972; Schamel et al., 2004). However, we did not detect significant within‐season shortening in incubation duration of phalaropes, which has been shown in other populations (Hötker, 1998; Reynolds, 1987; Schamel, 2000).

With the climate cooling scenario, our hypothesis would have predicted that delayed egg‐laying leads to smaller clutches, smaller eggs, shorter incubation duration, and lower egg survival (Figure 1b). However, despite significant delays in timing of breeding, none of the reproductive traits that exhibited within‐season trends also showed significant differences in the population means between decades. A 4‐ to 5‐day delay in egg‐laying was not enough to change the population means for clutch size or egg volume. Given the within‐season trends we found with egg volume, for example, a 5‐day delay in breeding should decrease the egg volume only by −0.1 ml and −0.005 ml for western and semipalmated sandpipers, respectively, and increase the egg volume of red‐necked phalaropes by +0.05 ml. Instead, two reproductive traits that did not show within‐season trends changed between decades. First, the daily nest survival rate of western sandpipers was lower in the 2010s than the 1990s (but with no net effect on the annual nest survival rate, given the change in incubation duration). Second, incubation duration between the decades decreased by 2 days for the sandpipers, but increased by 2 days for the phalaropes. Incubation duration is noteworthy as the only reproductive trait that we measured that is directly constrained by the total available length of the breeding season, hence the timing of the fall migration and scheduling of prebasic molt.

### 4.3 Change of incubation duration under climate cooling

The rate of embryonic development of a bird species may evolve to balance the life‐history trade‐off between mortality of the incubating adult and time‐dependent mortality risk to the eggs (Martin, 2002). Incubation duration is proximally adjusted by the foraging conditions for incubating adults, nest attentiveness, and microclimate conditions in the nest (Boersma, 1982). Both sandpiper species in our study provide biparental incubation and attend their clutch with high constancy (Bulla, Valcu, Rutten, & Kempenaers, 2014; Bulla et al., 2016), and ambient temperature during the incubation stage was similar between the two decades (Kwon, 2016). One possible mechanism for a biparental species with near‐constant nest attentiveness to shorten the incubation period is through adjusting the heat influx to their eggs while incubating, which has been shown for Arctic‐breeding sanderling (*C. alba*, Reneerkens, Grond, Schekkerman, Tulp, & Piersma, 2011).

If parents are increasing the heat influx to shorten the incubation duration, it is likely at an energetic expense compensated by potential advantages. A shorter incubation period reduces the number of days the eggs are exposed to potential predation risks. Within‐season increases in predation risk and the chance of severe rain events toward the end of the breeding season also likely increase the benefit of a shortened incubation period, assuming that the phenology of predators has not changed or has been delayed at a similar rate (Schamel & Tracy, 1991). Delays in timing of clutch initiation in the 2010s could have favored a shortening of incubation so that breeders could meet optimal timing for departure on southbound migration. In both sandpiper species, females leave the breeding grounds before males (Butler, Kaiser, & Smith, 1987). Western sandpipers breeding later in the season have shortened parental care and stopover duration to reduce predation risk at stopover sites, which suggests there may be a temporal threshold for successful fall migration for sandpipers (Jamieson et al., 2014; Lank et al., 2003; Ydenberg, Butler, Lank, Smith, & Ireland, 2004). If one parent departs more than a few days before the nest hatches, the clutch typically fails (Erckmann, 1981; but see Bulla et al., 2017), so advances in hatching dates may allow parents to successfully hatch a late nest while still meeting the optimal timing for departure on fall migration.

Temperature is not just an environmental cue for timing of breeding in birds, but can also directly influence various aspects of breeding (Stevenson & Bryant, 2000). Energetic costs of incubation may not affect the incubation schedule of the biparental semipalmated sandpipers (Bulla, Cresswell, Rutten, Valcu, & Kempenaers, 2015), but may affect the incubation of uniparental male phalaropes. Red‐necked phalaropes at Nome incubated only about 70% of the time (English, 2014) and tended to have longer incubation durations during the early stages of a breeding season when the ambient temperatures were relatively low (English, 2014; Reynolds, 1987; Schamel, 2000). At Nome, nest attentiveness of phalaropes is lower with colder weather, larger egg sizes, and poorer body condition of incubating males (English, 2014). These patterns suggest that self‐maintenance directly trades off against incubation, and we would expect incubation duration to vary as a result of changes in incubation behavior. Furthermore, our finding of a within‐season increase in the egg volume of phalaropes, as opposed to the within‐season decreases seen in sandpipers, suggests that colder temperatures early in the breeding season either directly limit energetic investment by female phalaropes through limited food availability, or indirectly select for females laying smaller eggs earlier in the season to maximize the number of clutches she lays per season or match the more limited incubation capabilities of small‐bodied males (English, 2014; Lislevand & Thomas, 2006). Red‐necked phalaropes tended to nest in wetter habitats than the other two sandpiper species breeding at our site, but the absolute distance among nests of three species was often close as within few meters, and nests likely experienced similar microclimates. A small sample size of only five phalarope nests in the 1990s allows only tentative conclusions, but the incubation duration of phalaropes appears to respond in the opposite direction from the biparental species, possibly due to different energetic costs. The pattern deserves further study in other species of shorebirds with uniparental and biparental care.

Our study shows that season‐specific climatic trends may not match annual averages and that local patterns may not match the global trends of climate warming. Three sympatric species of shorebirds at Nome, Alaska, all delayed clutch initiation at a similar rate as the local climate cooled. Reproductive traits such as clutch size, egg volume, and daily nest survival generally did not vary with delayed breeding between decades. However, delayed breeding was coupled with decadal changes in incubation duration, with the direction of changes differing among species with different incubation systems. Our results indicate that the breeding phenology of shorebirds can closely track interannual variation in temperature conditions at their breeding grounds, and the effects can vary among reproductive traits and among sympatric species. Furthermore, delayed breeding and shortened incubation duration provides evidence for the hypothesis that Arctic‐breeding species are temporally constrained. Further research to test a temporal constraint of Arctic breeding could include experimentally delaying clutch initiation and assessing the impacts on incubation duration.

## CONFLICT OF INTEREST

None declared.

## AUTHOR CONTRIBUTIONS

EK, BKS, and DBL conceived the ideas and designed methodology; EK analyzed the data and led the writing of the manuscript. All authors collected field data and contributed critically to drafts of the manuscript.

## DATA ACCESSIBILITY

All data used in our analyses are available online at the NSF Arctic Data Center: https://arcticdata.io/catalog/#view/doi:10.18739/A2CD5M.

## References

[ece33733-bib-0002] AMAP . (2012). Arctic Climate Issues 2011: Changes in Arctic snow, water, ice and permafrost. SWIPA 2011. Overview Report. 112 pp.

[ece33733-bib-0004] Bates, D. , Maechler, M. , Bolker, B. , & Walker, S. (2014). lme4: Linear mixed‐effects models using Eigen and S4. R package version 1.1.

[ece33733-bib-0005] Birkhead, T. R. , & Nettleship, D. N. (1982). The adaptive significance of egg size and laying date in thick‐billed murres *Uria lomvia* . Ecology, 63, 300–306. https://doi.org/10.2307/1938946

[ece33733-bib-0006] Boersma, P. D. (1982). Why some birds take so long to hatch. American Naturalist, 120, 733–750. https://doi.org/10.1086/284027

[ece33733-bib-0007] Borboroglu, P. G. , Yorio, P. A. , Moreno, J. U. , & Potti, J. A. (2008). Seasonal decline in breeding performance of the kelp gull *Larus dominicanus* . Marine Ornithology, 36, 153–157.

[ece33733-bib-0008] Both, C. , Artemyev, A. V. , Blaauw, B. , Cowie, R. J. , Dekhuijzen, A. J. , Eeva, T. , … Metcalfe, N. B. (2004). Large‐scale geographical variation confirms that climate change causes birds to lay earlier. Proceedings of the Royal Society of London B, 271, 1657–1662. https://doi.org/10.1098/rspb.2004.2770 10.1098/rspb.2004.2770 PMC1691776 15306284

[ece33733-bib-0009] Both, C. , & Visser, M. E. (2005). The effect of climate change on the correlation between avian life‐history traits. Global Change Biology, 11, 1606–1613. https://doi.org/10.1111/j.1365-2486.2005.01038.x

[ece33733-bib-0010] Brown, J. L. , Li, S. H. , & Bhagabati, N. (1999). Long‐term trend toward earlier breeding in an American bird: A response to global warming? Proceedings of the National Academy of Sciences USA, 96, 5565–5569. https://doi.org/10.1073/pnas.96.10.5565 10.1073/pnas.96.10.5565 PMC21900 10318924

[ece33733-bib-0011] Bulla, M. , Cresswell, W. , Rutten, A. L. , Valcu, M. , & Kempenaers, B. (2015). Biparental incubation‐scheduling: No experimental evidence for major energetic constraints. Behavioral Ecology, 26, 30–37. https://doi.org/10.1093/beheco/aru156 25713473 10.1093/beheco/aru156 PMC4309980

[ece33733-bib-0012] Bulla, M. , Prüter, H. , Vitnerová, H. , Tijsen, W. , Sládeček, M. , Alves, J. A. , Gilg, O. , Kempenaers, B. (2017). Flexible parental care: Uniparental incubation in biparentally incubating shorebirds. Scientific Reports, 7, 12851 https://doi.org/10.1038/s41598-017-13005-y 29038493 10.1038/s41598-017-13005-y PMC5643509

[ece33733-bib-0013] Bulla, M. , Valcu, M. , Dokter, A. M. , Dondua, A. G. , Kosztolányi, A. , Rutten, A. L. , … Spiegel, C. S. (2016). Unexpected diversity in socially synchronized rhythms of shorebirds. Nature, 540, 109–113. https://doi.org/10.1038/nature20563 27880762 10.1038/nature20563

[ece33733-bib-0014] Bulla, M. , Valcu, M. , Rutten, A. L. , & Kempenaers, B. (2014). Biparental incubation patterns in a high‐Arctic breeding shorebird: How do pairs divide their duties? Behavioral Ecology, 25, 152–164. https://doi.org/10.1093/beheco/art098 24347997 10.1093/beheco/art098 PMC3860833

[ece33733-bib-0015] Burnham, K. P. , & Anderson, D. R. (2002). Model selection and multimodel inference: A practical information‐theoretic approach. New York, NY: Springer Science & Business Media.

[ece33733-bib-0016] Butler, R. , Kaiser, G. W. , & Smith, G. E. J. (1987). Migration chronology, length of stay, sex ratio, and weight of western sandpipers, (*Calidris mauri*) on the south coast of British Columbia. Journal of Field Ornithology, 58, 103–111.

[ece33733-bib-0018] Cahill, A. E. , Aiello‐Lammens, M. E. , Fisher‐Reid, M. C. , Hua, X. , Karanewsky, C. J. , Ryu, H. Y. , … Wiens, J. J. (2013). How does climate change cause extinction? Proceedings of the Royal Society of London B, 280, 20121890. 10.1098/rspb.2012.1890 PMC3574421 23075836

[ece33733-bib-0019] Charmantier, A. , McCleery, R. H. , Cole, L. R. , Perrins, C. , Kruuk, L. E. , & Sheldon, B. C. (2008). Adaptive phenotypic plasticity in response to climate change in a wild bird population. Science, 320, 800–803. https://doi.org/10.1126/science.1157174 18467590 10.1126/science.1157174

[ece33733-bib-0020] Clausen, K. K. , & Clausen, P. (2013). Earlier springs cause phenological mismatch in long‐distance migrants. Oecologia, 173, 1101–1112. https://doi.org/10.1007/s00442-013-2681-0 23660701 10.1007/s00442-013-2681-0

[ece33733-bib-0022] Crick, H. Q. , Dudley, C. , Glue, D. E. , & Thomson, D. L. (1997). UK birds are laying eggs earlier. Nature, 388, 526 https://doi.org/10.1038/41453

[ece33733-bib-0023] Crick, H. Q. P. , Gibbons, D. W. , & Magrath, R. D. (1993). Seasonal changes in clutch size in British birds. Journal of Animal Ecology, 62, 263–273. https://doi.org/10.2307/5357

[ece33733-bib-0024] Crick, H. Q. P. , & Sparks, T. H. (1999). Climate change related to egg‐laying trends. Nature, 399, 423–424. https://doi.org/10.1038/20839

[ece33733-bib-0025] Dickey, M. H. , Gauthier, G. , & Cadieux, M.‐C. (2008). Climate effects on the breeding phenology and reproductive success of an Arctic‐nesting goose species. Global Change Biology, 14, 1973–1985. https://doi.org/10.1111/j.1365-2486.2008.01622.x

[ece33733-bib-0026] Doran, P. T. , Priscu, J. C. , Lyons, W. B. , Walsh, J. E. , Fountain, A. G. , McKnight, D. M. , … Fritsen, C. H. (2002). Antarctic climate cooling and terrestrial ecosystem response. Nature, 415, 517–520. https://doi.org/10.1038/nature710 11793010 10.1038/nature710

[ece33733-bib-0027] Dunn, P. O. , & Winkler, D. W. (1999). Climate change has affected the breeding date of tree swallows throughout North America. Proceedings of the Royal Society of London B, 266, 2487–2490. https://doi.org/10.1098/rspb.1999.0950 10.1098/rspb.1999.0950 PMC1690485 10693819

[ece33733-bib-0028] English, W. B. (2014). The evolutionary ecology of reproductive traits in the red‐necked phalarope (Phalaropus lobatus). MS thesis. Burnaby, BC: Simon Fraser University.

[ece33733-bib-0029] English, W. B. , Schamel, D. , Tracy, D. M. , Westneat, D. F. , & Lank, D. B. (2014). Sex ratio varies with egg investment in the red‐necked phalarope (*Phalaropus lobatus*). Behavioral Ecology and Sociobiology, 68, 1939–1949. https://doi.org/10.1007/s00265-014-1800-1

[ece33733-bib-0030] Erckmann Jr., W. J. (1981). The evolution of sex‐role reversal and monogamy in shorebirds. PhD Thesis. Seattle, WA: University of Washington.

[ece33733-bib-0031] Franks, S. , David, B. L. , & Herbert, W. (2014). Western sandpiper (*Calidris mauri*) In Rodewald A. (Ed.), The birds of North America Online. Ithaca, NY: Cornell Lab of Ornithology Retrieved from the Birds of North America Online: http://bna.birds.cornell.edu/bna/species/090

[ece33733-bib-0032] Gelman, A. , & Su, Y.S. (2015). arm: Data analysis using regression and multilevel/hierarchical models. R package version 1.8‐5. http://CRAN.R-project.org/package=arm.

[ece33733-bib-0033] Governali, F. C. , Gates, H. R. , Lanctot, R. B. , & Holmes, R. T. (2012). Egg volume can be accurately and efficiently estimated from linear dimensions for Arctic‐breeding shorebirds. Wader Study Group Bulletin, 119, 46–51.

[ece33733-bib-0034] Grant, T. A. , Shaffer, T. L. , Madden, E. M. , & Pietz, P. J. (2005). Time‐specific variation in passerine nest survival: New insights into old questions. Auk, 122, 661–672. https://doi.org/10.1642/0004-8038(2005)122[0661:TVIPNS]2.0.CO;2

[ece33733-bib-0035] Gurney, K. E. , Clark, R. G. , Slattery, S. M. , Smith‐Downey, N. V. , Walker, J. , Armstrong, L. M. , … DeGroot, K. A. (2011). Time constraints in temperate‐breeding species: Influence of growing season length on reproductive strategies. Ecography, 34, 628–636. https://doi.org/10.1111/j.1600-0587.2010.06622.x

[ece33733-bib-0036] Hargitai, R. , Herényi, M. , Nagy, G. , Nyiri, Z. , Eke, Z. , & Török, J. (2016). Effects of environmental conditions on the egg mass, yolk antioxidant level, eggshell thickness and eggshell spotting patterns of great tits (*Parus major*). Journal of Ornithology, 157, 995–1006. https://doi.org/10.1007/s10336-016-1348-0

[ece33733-bib-0037] Hicklin, P. , & Gratto‐Trevor, C. L. (2010). Semipalmated sandpiper (*Calidris pusilla*) In Rodewald A. (Ed.), The birds of North America Online. Ithaca, NY: Cornell Lab of Ornithology Retrieved from the Birds of North America Online: http://bna.birds.cornell.edu/bna/species/006

[ece33733-bib-0038] Hildén, O. , & Vuolanto, S. (1972). Breeding biology of the red‐necked phalarope *Phalaropus lobatus* in Finland. Ornis Fennica, 49, 57–85.

[ece33733-bib-0039] Hipfner, J. M. , Gaston, A. J. , & Gilchrist, H. G. (2005). Variation in egg size and laying date in thick‐billed murre populations breeding in the low Arctic and high Arctic. Condor, 107, 657–664. https://doi.org/10.1650/0010-5422(2005)107[0657:VIESAL]2.0.CO;2

[ece33733-bib-0040] Hötker, H. (1998). Intraspecific variation in length of incubation period in avocets *Recurvirostra avosetta* . Ardea, 86, 33–41.

[ece33733-bib-0041] Høye, T. T. , Post, E. , Meltofte, H. , Schmidt, N. M. , & Forchhammer, M. C. (2007). Rapid advancement of spring in the high Arctic. Current Biology, 17, 449–451. https://doi.org/10.1016/j.cub.2007.04.047 10.1016/j.cub.2007.04.047 17580070

[ece33733-bib-0042] IPCC . (2014). Climate change 2014: Impacts, adaptation, and vulnerability. Part B: Regional aspects In Barros V. R., Field C. B., Dokken D. J., Mastrandrea M. D., Mach K. J., Bilir T. E., Chatterjee M., Ebi K. L., Estrada Y. O., Genova R. C., Girma B., Kissel E. S., Levy A. N., MacCracken S., Mastrandrea P. R., & White L. L. (Eds.), Contribution of working group II to the fifth assessment report of the Intergovernmental Panel on Climate Change (p. 688). Cambridge, UK: Cambridge University Press.

[ece33733-bib-0043] Jamieson, S. E. , Ydenberg, R. C. , & Lank, D. B. (2014). Does predation danger on southward migration curtail parental investment by female western sandpipers? Animal Migration, 2, 34–43.

[ece33733-bib-0044] Johnson, M. , & Walters, J. R. (2008). Effects of mate and site fidelity on nest survival of western sandpipers (*Calidris mauri*). Auk, 125, 76–86. https://doi.org/10.1525/auk.2008.125.1.76

[ece33733-bib-0046] Kwon, E. (2016). Effects of climate change on the breeding ecology and trophic interactions of Arctic‐breeding shorebirds. PhD Thesis. Manhattan, KS: Kansas State University.

[ece33733-bib-0047] Laake, J.L. (2013). RMark: An R interface for analysis of capture‐recapture data with MARK. AFSC Processed Rep 2013‐01, 25p. Seattle, WA: Alaska Fish. Sci. Cent., NOAA, Natl. Mar. Fish. Serv.

[ece33733-bib-0048] Lank, D. B. , Butler, R. W. , Ireland, J. , & Ydenberg, R. C. (2003). Effects of predation danger on migration strategies of sandpipers. Oikos, 103, 303–319. https://doi.org/10.1034/j.1600-0706.2003.12314.x

[ece33733-bib-0049] Lank, D. B. , Oring, L. W. , & Maxson, S. J. (1985). Mate and nutrient limitation of egg‐laying in a polyandrous shorebird. Ecology, 66, 1513–1524. https://doi.org/10.2307/1938014

[ece33733-bib-0050] Lepage, D. , Gauthier, G. , & Menu, S. (2000). Reproductive consequences of egg‐laying decisions in snow geese. Journal of Animal Ecology, 69, 414–427. https://doi.org/10.1046/j.1365-2656.2000.00404.x

[ece33733-bib-0051] Liebezeit, J. R. , Gurney, K. E. , Budde, M. , Zack, S. , & Ward, D. (2014). Phenological advancement in arctic bird species: Relative importance of snow melt and ecological factors. Polar Biology, 37, 1309–1320. https://doi.org/10.1007/s00300-014-1522-x

[ece33733-bib-0052] Liebezeit, J. R. , Smith, P. A. , Lanctot, R. B. , Schekkerman, H. , Tulp, I. , Kendall, S. J. , … Gratto‐Trevor, C. (2007). Assessing the development of shorebird eggs using the flotation method: Species‐specific and generalized regression models. Condor, 109, 32–47. https://doi.org/10.1650/0010-5422(2007)109[32:ATDOSE]2.0.CO;2

[ece33733-bib-0053] Lislevand, T. , & Thomas, G. H. (2006). Limited male incubation availability and the evolution of egg size in shorebirds. Biology Letters, 2, 206–208. https://doi.org/10.1098/rsbl.2005.0428 17148363 10.1098/rsbl.2005.0428 PMC1618901

[ece33733-bib-0054] Martin, T. E. (2002). A new view of avian life‐history evolution tested on an incubation paradox. Proceedings of the Royal Society of London B, 269, 309–316. https://doi.org/10.1098/rspb.2001.1879 10.1098/rspb.2001.1879 PMC1690888 11839200

[ece33733-bib-0055] McCarty, J. P. (2001). Ecological consequences of recent climate change. Conservation Biology, 15, 320–331. https://doi.org/10.1046/j.1523-1739.2001.015002320.x

[ece33733-bib-0056] McCleery, R. H. , & Perrins, C. M. (1998). Temperature and egg‐laying trends. Nature, 391, 30–31. https://doi.org/10.1038/34073

[ece33733-bib-0057] McKinnon, L. , Picotin, M. , Bolduc, E. , Juillet, C. , & Bêty, J. (2012). Timing of breeding, peak food availability, and effects of mismatch on chick growth in birds nesting in the High Arctic. Canadian Journal of Zoology, 90, 961–971. https://doi.org/10.1139/z2012-064

[ece33733-bib-0058] Mortensen, L. O. , Schmidt, N. M. , Høye, T. T. , Damgaard, C. , & Forchhammer, M. C. (2016). Analysis of trophic interactions reveals highly plastic response to climate change in a tri‐trophic High‐Arctic ecosystem. Polar Biology, 39, 1467–1478. https://doi.org/10.1007/s00300-015-1872-z

[ece33733-bib-0500] Nakagawa, S. , & Freckleton, R. P. (2011). Model averaging, missing data and multiple imputation: a case study for behavioural ecology. Behavioral Ecology and Sociobiology, 65, 103–116. https://doi.org/10.1007/s00265-010-1044-7

[ece33733-bib-0059] Nol, E. , Blanken, M. S. , & Flynn, L. (1997). Sources of variation in clutch size, egg size and clutch completion dates of semipalmated plovers in Churchill, Manitoba. Condor, 99, 389–396. https://doi.org/10.2307/1369945

[ece33733-bib-0060] O'Brien, R. M. (2007). A caution regarding rules of thumbs for variance inflation factors. Quality & Quantity, 41, 673–690. https://doi.org/10.1007/s11135-006-9018-6

[ece33733-bib-0061] Ockendon, N. , Leech, D. , & Pearce‐Higgins, J. W. (2013). Climatic effects on breeding grounds are more important drivers of breeding phenology in migrant birds than carry‐over effects from wintering grounds. Biology Letters, 9, 20130669 https://doi.org/10.1098/rsbl.2013.0669 24196517 10.1098/rsbl.2013.0669 PMC3871353

[ece33733-bib-0062] Parmesan, C. (2006). Ecological and evolutionary responses to recent climate change. Annual Review of Ecology, Evolution, and Systematics, 37, 637–669. https://doi.org/10.1146/annurev.ecolsys.37.091305.110100

[ece33733-bib-0063] Parmesan, C. , & Yohe, G. (2003). A globally coherent fingerprint of climate change impacts across natural systems. Nature, 421, 37–42. https://doi.org/10.1038/nature01286 12511946 10.1038/nature01286

[ece33733-bib-0064] Perrins, C. M. (1970). The timing of birds’ breeding seasons. Ibis, 112, 242–255.

[ece33733-bib-0065] Pinheiro, J. , Bates, D. , DebRoy, S. , Sarkar, D. , & R Core Team . (2017). nlme: Linear and Nonlinear Mixed Effects Models. R package version 3.1‐131, https://CRAN.R-project.org/package=nlme.

[ece33733-bib-0067] Powell, L. A. (2007). Approximating variance of demographic parameters using the delta method: A reference for avian biologists. Condor, 109, 949–954. https://doi.org/10.1650/0010-5422(2007)109[949:AVODPU]2.0.CO;2

[ece33733-bib-0068] R Development Core Team . (2016). R: A language and environment for statistical computing. Vienna, Austria: R Foundation for Statistical Computing Available at: https://www.R-project.org/.

[ece33733-bib-0069] Reneerkens, J. , Grond, K. , Schekkerman, H. , Tulp, I. , & Piersma, T. (2011). Do uniparental sanderlings *Calidris alba* increase egg heat input to compensate for low nest attentiveness? PLoS ONE, 6, e16834 https://doi.org/10.1371/journal.pone.0016834 21347377 10.1371/journal.pone.0016834 PMC3036718

[ece33733-bib-0070] Reneerkens, J. , Schmidt, N. M. , Gilg, O. , Hansen, J. , Hansen, L. H. , Moreau, J. , & Piersma, T. (2016). Effects of food abundance and early clutch predation on reproductive timing in a high Arctic shorebird exposed to advancements in arthropod abundance. Ecology and Evolution, 6, 7375–7386. https://doi.org/10.1002/ece3.2361 28725405 10.1002/ece3.2361 PMC5513252

[ece33733-bib-0071] Reynolds, J. D. (1987). Mating system and nesting biology of the red‐necked phalarope *Phalaropus lobatus*: What constrains polyandry? Ibis, 129, 225–242.

[ece33733-bib-0072] Royle, N. J. , Hartley, I. R. , & Parker, G. A. (2006). Consequences of biparental care for begging and growth in zebra finches, *Taeniopygia guttata* . Animal Behaviour, 72, 123–130. https://doi.org/10.1016/j.anbehav.2005.09.023

[ece33733-bib-0073] Ruthrauff, D. R. (2002). Seasonal and age‐related trends in the reproductive output of western sandpipers (Calidris mauri) at Kanaryaraq, Alaska. MS Thesis. Arcadia, CA: Humboldt State University.

[ece33733-bib-0074] Sandercock, B. K. (1997a). Incubation capacity and clutch size determination in two calidrine sandpipers: A test of the four‐egg threshold. Oecologia, 110, 50–59. https://doi.org/10.1007/s004420050132 28307468 10.1007/s004420050132

[ece33733-bib-0075] Sandercock, B. K. (1997b). The breeding biology of red‐necked phalaropes *Phalaropus lobatus* at Nome, Alaska. Wader Study Group Bulletin, 83, 50–54.

[ece33733-bib-0076] Sandercock, B. K. (1998). Chronology of nesting events in western and semipalmated sandpipers near the Arctic circle. Journal of Field Ornithology, 69, 235–243.

[ece33733-bib-0077] Sandercock, B. K. , Lank, D. B. , & Cooke, F. (1999). Seasonal declines in the fecundity of Arctic‐breeding sandpipers: Different tactics in two species with an invariant clutch size. Journal of Avian Biology, 30, 460–468. https://doi.org/10.2307/3677018

[ece33733-bib-0078] Schamel, D. (2000). Female and male reproductive strategies in the red‐necked phalarope, a polyandrous shorebird. PhD Thesis. Burnaby, BC: Simon Fraser University.

[ece33733-bib-0079] Schamel, D. , & Tracy, D. M. (1991). Breeding site fidelity and natal philopatry in the sex role‐reversed red and red‐necked phalaropes. Journal of Field Ornithology, 62, 390–398.

[ece33733-bib-0080] Schamel, D. , Tracy, D. M. , & Lank, D. B. (2004). Male mate choice, male availability and egg production as limitations on polyandry in the red‐necked phalarope. Animal Behaviour, 67, 847–853. https://doi.org/10.1016/j.anbehav.2003.04.014

[ece33733-bib-0081] Skagen, S. K. , & Adams, A. A. Y. (2012). Weather effects on avian breeding performance and implications of climate change. Ecological Applications, 22, 1131–1145. https://doi.org/10.1890/11-0291.1 22827123 10.1890/11-0291.1

[ece33733-bib-0082] Skinner, W. R. , Jefferies, R. L. , Carleton, T. J. , & Abraham, R. F. (1998). Prediction of reproductive success and failure in lesser snow geese based on early season climatic variables. Global Change Biology, 4, 3–16. https://doi.org/10.1046/j.1365-2486.1998.00097.x

[ece33733-bib-0083] Slater, F. M. (1999). First egg date fluctuations for the pied flycatcher *Ficedula hypoleuca* in the woodlands of mid‐Wales in the twentieth century. Ibis, 141, 489–506.

[ece33733-bib-0084] Stenseth, N. C. , & Mysterud, A. (2002). Climate, changing phenology, and other life history traits: Nonlinearity and match–mismatch to the environment. Proceedings of the National Academy of Sciences USA, 99, 13379–13381. https://doi.org/10.1073/pnas.212519399 10.1073/pnas.212519399 PMC129680 12370424

[ece33733-bib-0085] Stevenson, I. R. , & Bryant, D. M. (2000). Avian phenology: Climate change and constraints on breeding. Nature, 406, 366–367. https://doi.org/10.1038/35019151 10935624 10.1038/35019151

[ece33733-bib-0086] Te Marvelde, L. , Webber, S. L. , Meijer, H. A. , & Visser, M. E. (2011). Mismatched reproduction is energetically costly for chick feeding female great tits. Functional Ecology, 25, 1302–1308. https://doi.org/10.1111/j.1365-2435.2011.01889.x

[ece33733-bib-0087] Tulp, I. , & Schekkerman, H. (2008). Has prey availability for Arctic birds advanced with climate change? Hindcasting the abundance of tundra arthropods using weather and seasonal variation. Arctic, 61, 48–60.

[ece33733-bib-0088] Verhulst, S. , & Nilsson, J.‐Å. (2008). The timing of birds’ breeding seasons: A review of experiments that manipulated timing of breeding. Philosophical Transactions of the Royal Society B, 363, 399–410. https://doi.org/10.1098/rstb.2007.2146 10.1098/rstb.2007.2146 PMC2606757 17666390

[ece33733-bib-0089] White, G. C. , & Burnham, K. P. (1999). Program MARK: Survival estimation from populations of marked animals. Bird Study, 46(Supplement), 120–138. https://doi.org/10.1080/00063659909477239

[ece33733-bib-0090] Winkler, D. W. , Dunn, P. O. , & McCulloch, C. E. (2002). Predicting the effects of climate change on avian life‐history traits. Proceedings of the National Academy of Sciences USA, 99, 13595–13599. https://doi.org/10.1073/pnas.212251999 10.1073/pnas.212251999 PMC129719 12370441

[ece33733-bib-0091] Ydenberg, R. C. , Butler, R. W. , Lank, D. B. , Smith, B. D. , & Ireland, J. (2004). Western sandpipers have altered migration tactics as peregrine falcon populations have recovered. Proceedings of the Royal Society of London B, 271, 1263–1269. https://doi.org/10.1098/rspb.2004.2713 10.1098/rspb.2004.2713 PMC1691718 15306350

