# Supplementary material for: Delayed egg‐laying and shortened incubation duration of Arctic‐breeding shorebirds coincide with climate cooling
Source: Ecol Evol. 2017 Dec 25;8(2):1339–51. doi: 10.1002/ece3.3733 (PMC5773331; doi:10.1002/ece3.3733)
Supplement: Supplementary file 1 [file ECE3-8-1339-s001.docx]

**Supplementary Table 1.** A set of models compared to test the effects of climatic covariates on timing of clutch initiation at Nome, Alaska, 1993−1996 and 2010−2014. Covariates included in the global model were average daily mean temperature during the pre-laying and egg-laying stages (‘Temp*_prelay_*’ and ‘Temp*_lay_*’), average daily total precipitation during the pre-laying and egg-laying stages (‘Rain*_prelay_*’ and ‘Rain*_lay_*’), the maximum snow accumulation during prior winter (‘Snow’), and species.

| Models | df | $\Delta$AIC*_c_* | weight |
| --- | --- | --- | --- |
| Species + Temp*_lay_* + Temp*_prelay_* | 7 | 0 | 0.371 |
| Species + Temp*_lay_* + Temp*_prelay_* + Rain*_lay_* | 8 | 1.80 | 0.151 |
| Species + Temp*_lay_* + Temp*_prelay_* + Rain*_prelay_* | 8 | 1.88 | 0.145 |
| Species + Temp*_lay_* + Temp*_prelay_* + Snow | 8 | 2.02 | 0.135 |
| Species + Temp*_lay_* + Temp*_prelay_* + Rain*_lay_* + Rain*_prelay_* | 9 | 3.69 | 0.059 |
| Species + Temp*_lay_* + Temp*_prelay_* + Rain*_lay_* + Snow | 9 | 3.81 | 0.055 |
| Species + Temp*_lay_* + Temp*_prelay_* + Rain*_prelay_* + Snow | 9 | 3.90 | 0.053 |
| Species + Temp*_lay_* + Temp*_prelay_* + Rain*_lay_* + Rain*_prelay_* + Snow | 10 | 5.71 | 0.021 |
| Species + Temp*_prelay_* + Rain*_lay_* | 7 | 8.90 | 0.004 |
| Species + Temp*_prelay_* + Rain*_lay_* + Snow | 8 | 10.51 | 0.002 |
| Species + Temp*_prelay_* + Rain*_lay_* + Rain*_prelay_* | 8 | 10.90 | 0.002 |
| Species + Temp*_prelay_* + Rain*_lay_* + Rain*_prelay_* + Snow | 9 | 12.49 | 0.001 |
| Species + Rain*_lay_* | 6 | 13.48 | 0 |
| Species + Rain*_lay_* + Rain*_prelay_* | 7 | 14.42 | 0 |
| Species + Rain*_lay_* + Snow | 7 | 14.89 | 0 |
| Species + Temp*_lay_* + Rain*_lay_* | 7 | 15.34 | 0 |
| Species + Rain*_lay_* + Rain*_prelay_* + Snow | 8 | 15.75 | 0 |
| Species + Temp*_prelay_* | 6 | 15.78 | 0 |
| Species + Temp*_prelay_* + Rain*_prelay_* | 7 | 15.89 | 0 |
| Species + Temp*_lay_* + Rain*_lay_* + Rain*_prelay_* | 8 | 16.44 | 0 |
| Species + Temp*_lay_* + Rain*_lay_* + Snow | 8 | 16.76 | 0 |
| Species + Temp*_prelay_* + Snow | 7 | 17.74 | 0 |
| Species + Temp*_lay_* + Rain*_lay_* + Rain*_prelay_* + Snow | 9 | 17.77 | 0 |
| Species + Temp*_prelay_* + Rain*_prelay_* + Snow | 8 | 17.86 | 0 |
| Species | 5 | 19.91 | 0 |
| Species + Temp*_lay_* | 6 | 20.30 | 0 |
| Species + Rain*_prelay_* | 6 | 21.07 | 0 |
| Species + Snow | 6 | 21.71 | 0 |
| Species + Temp*_lay_* + Snow | 7 | 22.05 | 0 |
| Species + Temp*_lay_* + Rain*_prelay_* | 7 | 22.32 | 0 |
| Species + Rain*_prelay_* + Snow | 7 | 22.88 | 0 |
| Species + Temp*_lay_* + Rain*_prelay_* + Snow | 8 | 24.07 | 0 |
| Temp*_lay_* + Temp*_prelay_* + Rain*_lay_* | 6 | 91.78 | 0 |
| Temp*_lay_* + Temp*_prelay_* + Rain*_lay_* + Snow | 7 | 93.34 | 0 |
| Temp*_lay_* + Temp*_prelay_* + Rain*_lay_* + Rain*_prelay_* | 7 | 93.68 | 0 |
| Temp*_lay_* + Temp*_prelay_* + Rain*_lay_* + Rain*_prelay_* + Snow | 8 | 95.21 | 0 |
| Temp*_lay_* + Temp*_prelay_* + Rain*_prelay_* | 6 | 111.72 | 0 |
| Temp*_lay_* + Temp*_prelay_* + Rain*_prelay_* + Snow | 7 | 113.54 | 0 |
| Temp*_lay_* + Temp*_prelay_* | 5 | 115.32 | 0 |
| Temp*_lay_* + Temp*_prelay_* + Snow | 6 | 117.10 | 0 |
| Temp*_prelay_* + Rain*_lay_* + Rain*_prelay_* | 6 | 135.99 | 0 |
| Temp*_prelay_* + Rain*_lay_* + Rain*_prelay_* + Snow | 7 | 137.34 | 0 |
| Temp*_prelay_* + Rain*_prelay_* | 5 | 152.14 | 0 |
| Temp*_prelay_* + Rain*_prelay_* + Snow | 6 | 153.83 | 0 |
| Temp*_prelay_* | 4 | 166.25 | 0 |
| Temp*_prelay_* + Snow | 5 | 167.93 | 0 |
| Temp*_prelay_* + Rain*_lay_* | 5 | 168.20 | 0 |
| Temp*_prelay_* + Rain*_lay_* + Snow | 6 | 169.87 | 0 |
| Temp*_lay_* + Rain*_lay_* | 5 | 215.00 | 0 |
| Temp*_lay_* + Rain*_lay_* + Rain*_prelay_* | 6 | 215.92 | 0 |
| Temp*_lay_* + Rain*_lay_* + Snow | 6 | 216.79 | 0 |
| Temp*_lay_* + Rain*_lay_* + Rain*_prelay_* + Snow | 7 | 217.66 | 0 |
| Temp*_lay_* + Rain*_prelay_* | 5 | 253.58 | 0 |
| Temp*_lay_* + Rain*_prelay_* + Snow | 6 | 255.56 | 0 |
| Temp*_lay_* | 4 | 257.80 | 0 |
| Temp*_lay_* + Snow | 5 | 259.78 | 0 |
| Rain*_lay_* + Rain*_prelay_* | 5 | 332.28 | 0 |
| Rain*_lay_* + Rain*_prelay_* + Snow | 6 | 333.97 | 0 |
| Rain*_prelay_* | 4 | 364.62 | 0 |
| Rain*_prelay_* + Snow | 5 | 366.52 | 0 |
| Rain*_lay_* | 4 | 460.07 | 0 |
| Rain*_lay_* + Snow | 5 | 462.02 | 0 |
| Null | 3 | 462.42 | 0 |
| Snow | 4 | 464.26 | 0 |
